# Does adipose tissue-derived stem cell therapy improve graft quality in freshly grafted ovaries?

**DOI:** 10.1186/s12958-015-0104-2

**Published:** 2015-09-23

**Authors:** Luciana L. Damous, Juliana S. Nakamuta, Ana ET Saturi de Carvalho, Katia Candido Carvalho, José Maria Soares-Jr, Manuel de Jesus Simões, José Eduardo Krieger, Edmund Chada Baracat

**Affiliations:** Laboratório de Ginecologia Estrutural e Molecular (LIM-58), Disciplina de Ginecologia, Departamento de Obstetrícia e Ginecologia, Hospital das Clínicas da Faculdade de Medicina da Universidade de São Paulo, Dr Arnaldo av 455, 2nd floor, room 2113, Pacaembu, 01246-903 São Paulo Brazil; Laboratory of Genetics and Molecular Cardiology, Heart Institute (Incor), Faculdade de Medicina da Universidade de São Paulo, Dr Enéas de Carvalho Aguiar Av 44, 10th floor, Cerqueira Cesar, 05403-000 São Paulo Brazil; Department of Morphology and Genetics, Universidade Federal de São Paulo (UNIFESP), Botucatu St 740. Ed. Lemos Torres, 2nd floor, Vila Clementino, 04023-009 São Paulo Brazil

**Keywords:** Ovarian transplantation, Fertility preservation, Cellular therapy, Stem cells, Ovary

## Abstract

**Background:**

A major concern in ovarian transplants is substantial follicle loss during the initial period of hypoxia. Adipose tissue-derived stem cells (ASCs) have been employed to improve angiogenesis when injected into ischemic tissue. This study evaluated the safety and efficacy of adipose tissue-derived stem cells (ASCs) therapy in the freshly grafted ovaries 30 days after injection.

**Methods:**

Rat ASCs (rASCs) obtained from transgenic rats expressing green fluorescent protein (GFP)-(5 × 10^4^ cells/ovary) were injected in topic (intact) or freshly grafted ovaries of 30 twelve-week-old adult female Wistar rats. The whole ovary was grafted in the retroperitoneum without vascular anastomosis, immediately after oophorectomy. Vaginal smears were performed daily to assess the resumption of the estrous cycle. Estradiol levels, grafts morphology and follicular viability and density were analyzed. Immunohistochemistry assays were conducted to identify and quantify rASC-GFP^+^, VEGF tissue expression, apoptosis (cleaved caspase-3 and TUNEL), and cell proliferation (Ki-67). Quantitative gene expression (qPCR) for VEGF-A, Bcl2, EGF and TGF-β1 was evaluated using RT-PCR and a double labeling immunofluorescence assay for GFP and Von Willebrand Factor (VWF) was performed.

**Results:**

Grafted ovaries treated with rASC-GFP^+^ exhibited earlier resumption of the estrous phase (*p* < 0.05), increased VEGF-A expression (11-fold in grafted ovaries and 5-fold in topic ovaries vs. control) and an increased number of blood vessels (*p* < 0.05) in ovarian tissue without leading to apoptosis or cellular proliferation (*p* > 0.05). Estradiol levels were similar among groups (*p* > 0.05). rASC-GFP^+^ were observed in similar quantities in the topic and grafted ovaries (*p* > 0.05), and double-labeling for GFP and vWF was observed in both injected groups.

**Conclusion:**

rASC therapy in autologous freshly ovarian grafts could be feasible and safe, induces earlier resumption of the estrous phase and enhances blood vessels in rats. This pilot study may be useful in the future for new researches on frozen-thawed ovarian tissue.

**Electronic supplementary material:**

The online version of this article (doi:10.1186/s12958-015-0104-2) contains supplementary material, which is available to authorized users.

## Background

Fertility preservation in patients who will undergo cytotoxic treatments is a matter of concern during therapeutic planning, and it is one of the foremost challenges of the next decade [1–4]. The most recent option for fertility preservation is the transplant of ovarian tissue, which has led to antral follicle development and live births [5–9].

Currently, more than 35 children born worldwide for whom this technique was used [5, 7, 8, 10], and the most attractive feature is the availability of thousands of oocytes in tissue strips, which enables prompt retrieval and fertilization [8–11]. Despite these successful outcomes, ovarian transplantation is still considered an experimental technique. The greatest concern is the short-term graft survival as a result of substantial follicle loss during transplantation [8, 9, 12].

Avascular ovarian grafts revascularize within 5 days after transplantation [13–15]. Numerous studies in animals and humans have previously demonstrated that the initial period of hypoxia is a determinant of the lifespan of the graft, and it is strongly negatively correlated with the success of ovarian transplants [13, 16–18]. Therefore, enhanced angiogenesis during this critical period could minimize ischemic effects, which is the main cause of follicular loss and ovarian injury. Several experimental models developed for this purpose have demonstrated promising results [19–26]. Specifically, cellular therapy has been shown to preserve hypoxic tissues, such as post infarction cardiac tissue. Important experimental findings in recent years suggest a considerable therapeutic potential for cellular replacement in the context of acute myocardial infarction and chronic, progressive cardiac disease [27–29].

Adipose tissue-derived stem cells (ASCs) have been employed in order to contribute to angiogenesis because these cells differentiate into or behave as endothelial cells after injection into ischemic tissue [30–32]. However, the mechanism of the beneficial effect is uncertain. ASCs exhibit a basal production of VEGF, which can be influenced by hypoxia [33]. The beneficial effects of ASC therapy on ischemic tissue may be mediated, at least in part, by paracrine factors [34]. However, the effects of cellular therapy on ovaries remain unclear.

ASCs enhance ovarian function when injected in rats with chemotherapy-damaged ovaries [35–37]. Though, little information is available regarding the therapeutic potential of an ovarian transplant. The aim of this study was to evaluate the effect of rat ASCs (rASCs) injected into topic or transplanted rat ovaries on regarding the safety and effects on fresh graft function. This may be the first step to evaluate cellular therapy on the ovarian tissue, for ensuring that these cells may not change ovary morphology.

## Methods

The study was performed at Laboratório de Ginecologia Estrutural e Molecular (LIM-58), Disciplina de Ginecologia, Departamento de Obstetrícia e Ginecologia, Hospital das Clínicas da Faculdade de Medicina da Universidade de São Paulo. (FMUSP), in cooperation with the Laboratory of Genetics and Molecular Cardiology/Heart Institute (Incor)/FMUSP. The Institutional Ethics Committee approved the experimental procedures, which followed the institutional guidelines for the care and use of laboratory animals (protocol 190/10).

The study sample consisted of 30 twelve-week-old adult female Wistar (*Rattus norvegicus albinus)* rats. The animals had access to a breed-specific food formula and water ad libitum throughout the experiment and were maintained under adequate sanitary, lighting (12/12 h), and temperature conditions in the animal laboratory.

### Experimental design

**Fig. 1:**
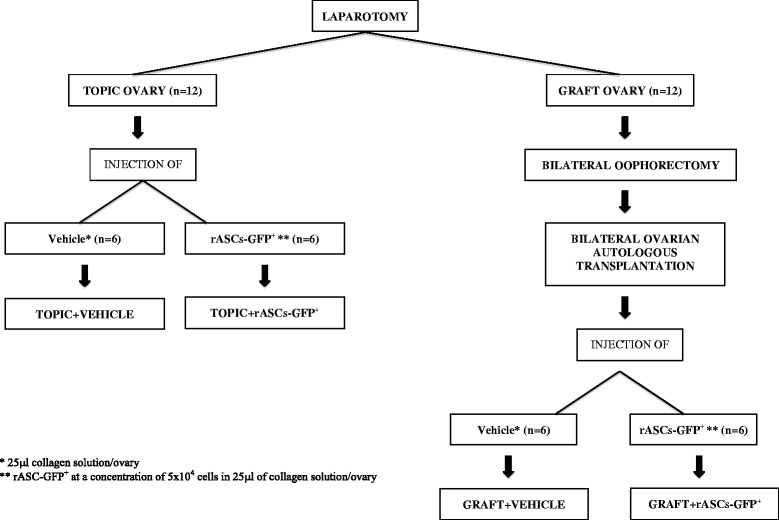
Experimental design

Vehicle or rASCs obtained from the inguinal region of Wistar rats transgenic for green fluorescent protein (GFP^+^) were injected into 24 Wistar rats. The rats were distributed in four experimental groups (Fig. 1): 1) Topic ovary + vehicle, 2) Topic ovary + rASC-GFP^+^, 3) Ovarian graft + vehicle, and 4) Ovarian graft + rASC-GFP^+^. A topic ovary is an intact ovary in which only a vehicle or rASCs injection was performed. An ovarian graft is the whole ovary grafted into the retroperitoneum without vascular anastomosis, immediately after oophorectomy. Collagen solution type I from rat tail (SIGM-C3867, Sigma-Aldrich, Inc.), 3 mg/ml, pH = 7.2, was used as the vehicle because previous studies have shown that the beneficial effects of ASCs could be enhanced by co-injection with biopolymers [28, 29]. An extra group (*n* = 6) was included to compare the morphology of topic ovary with no treatment or treated with vehicle in the same animal.

### Cell isolation and *ex vivo* expansion

Inguinal subcutaneous adipose tissue was collected under sterile conditions from 10-week-old male Wistar rats and rinsed with phosphate-buffered saline (PBS). rASCs were isolated, characterized, and maintained in culture as previously described [29]. The cells were used for experimental procedures until passage 3. Details are available in the Aditional file 1 (ASCs isolation and ex vivo expansion).

The morphological and replicative characteristics as well as the immunophenotype (CD90^+^, CD29^+^, CD44^+^, CD73^+^, CD31^−^, CD45^−^) of the ASCs have been previously described in our laboratory [38]. Although the characterization has been performed extensively in mouse adipose-derived stem cells (mASCs), our group conducted some immunophenotyping assays in the rat adipose-derived stem cells (rASCs). The percentage of CD90- and CD29-positive cells, the primary markers of a variety of various adult stem cells, was found to be approximatelly 90 % (92.65 and 98.89 %, respectively) in rASCs at the third culture passage (unpublished data from Nakamuta et al.).

### Vaginal smear collection

Vaginal smears were obtained daily pre-operatively from each rat between 8:00 a.m. and 10:00 a.m. Only the animals that exhibited at least two consecutive normal four-day vaginal estrous cycles were included in the experiment. Ovarian transplantation was performed during the diestrus phase. Beginning on day 4 postoperatively (PO), vaginal smears were performed daily until euthanasia, which was performed between days 30 and day 35, always when the rats were in the diestrous phase.

Vaginal smears were obtained with a swab soaked in physiological solution, placed on a standard slide and immediately fixed in absolute alcohol for staining using the Shorr-Harris technique. The smears were analyzed under a light microscope at 10× and 40× magnification [39].

### Ovarian transplantation and cellular therapy

The animals were anesthetized with an intraperitoneal injection of xylazine and ketamine at doses of 15 and 60 mg.kg^−1^ of body weight, respectively. The abdominopelvic cavity was opened, and the ovaries were identified. The ovarian pedicles were clamped and immediately ligated with 4–0 nylon suture. The fallopian tubes were resected with periovarian adipose tissue fragments.

Each animal in the transplanted groups (*n* = 12) received both autologous ovary transplants. Intact whole ovaries were implanted in the retroperitoneum in the proximity aorta and vena cava using a simple stitch of 4–0 nylon suture, without vascular anastomosis, each on one side of the psoas muscle.

At this point, the transplanted groups were distributed in according to the treatment, as follows (*n* = 6 for each group): vehicle (25 μl of collagen solution/ovary) or rASC-GFP^+^ (at a concentration of 5 × 10^4^ cells in 25 μl of vehicle/ovary). The technical standardization of ASCs injection was performed according to a pilot project in which we tested different vehicle volumes for cell delivery (ranging from 2 to 50 μl) using a surgical microscope (16×). The standard dose of 25 μl was the lowest in which no leakage was visually observed following the injection, without changes in ovary morphology and ASC-GFP^+^ were easily identified and quantified in ovarian stroma (data not shown). The cells were injected by a single shot into the center of the ovarian parenchyma in both grafts. In the topic groups (*n* = 12), the only procedure performed was the injection of vehicle or rASC-GFP^+^.

After the treatments, the wall closure was performed using a 5–0 nylon monofilament thread on two planes, including the peritoneum-aponeurotic muscle and the skin.

### Graft retrieval and histological preparation

The animals underwent a second surgical procedure between PO days 30 and 35, always in the diestrus phase. The abdominal cavity was opened, and the ovaries were macroscopically identified and assessed. The muscle bed was also assessed for vascularization and surrounding adhesions. The grafts were subsequently removed whole. Blood samples were collected from vena cava for serum estradiol levels assay.

One ovarian graft was immediately fixed in 4 % paraformaldehyde for at least 24 h. Following fixation, the ovaries were dehydrated, paraffin-embedded, serially sectioned at 5 μm, and mounted on glass microscope slides. Routine hematoxylin and eosin (HE) staining was performed for histological examination with light microscopy. The ovarian cortex was sectioned into two pieces of equal size, and five representative sections were selected. The other graft was placed in liquid nitrogen at −196 °C until real-time polymerase chain reaction (RT-PCR) was performed. Following this procedure, the animals were euthanized with a lethal dose of the previously used anesthetics.

### Serum estradiol levels assay

Blood samples were collected from the vena cava, then transferred to tubes and stored overnight at 4 °C. These samples were centrifuged at 4 °C, 5000 rpm in a Sorvall ST16R centrifuge with rotor model 75003629 (Thermo Scientific, Asheville, NC, United States) for 15 min to separate the solid components from the serum, which was stored at − 20 °C. Serum estradiol levels were assessed using ELISA kits (Ucsn Life Sciences Inc., Wuhan, Hubei, China) following the instructions of the manufacturer. Results are expressed in picogram per ml (pg/ml).

### Morphological and morphometric analyses

Morphological evaluation was achieved through descriptive analyses of the grafts. The assessment of follicular quality was based on the cell density, the presence or absence of pyknotic bodies, and the integrity of the basement membrane and oocyte. The follicles were classified as normal or degenerated according to these criteria, and only the former were characterized and quantified [40, 41].

Viable follicles were classified into three groups: immature follicles (including primordial, primary and preantral), antral and corpus lutem [40, 41]. Primordial follicle exhibited only an oocyte and a layer of squamous cells. Primary follicle exhibited an oocyte and one or more layers of cuboidal or prismatic cells but no antrum. Preantral follicle exhibited an oocyte and not an antrum. Mature follicles contained an oocyte with antrum. The corpus luteum exhibited intact luteal cells that contained a nucleus and cells surrounded by capillary blood vessels [18, 41–44]. To avoid counting the same follicle more than once, only individual follicles having an oocyte containing a nucleus were evaluated. Counting was performed in eight fields per section. The quantification of blood vessels (number/mm^2^) was also performed using a computerized images system to obtain and analyze eight fields per animal of HE stained ovarian tissue (400×). All images of sections were obtained using an image acquisition software system (Leica DM2500; LEICA, Wetzlar, Germany). Two independent investigators blinded to the experimental treatments performed all analyses under a microscope (LEICA).

### Immunofluorescence and immunohistochemistry assays

Immunohistochemistry assays were performed for the identification and quantification of rASC-GFP^+^ in the ovarian stroma, and double-labeling immunofluorescence was performed for GFP^+^ and the von Willebrand factor (vWF), which is a marker of endothelial cells [45], to assess the endothelial phenotype from injected rASC-GFP^+^.

Cross-sections embedded in paraffin were treated with antigenic exposure and blocked with 2 % casein in PBS. The tissue sections were incubated with anti-GFP antibody (1:500, ab6556, Abcam Inc., Cambrigde, MA, USA) overnight at 4 °C for the identification and counting of rASC-GFP^+^. The sections were incubated with a biotinylated rabbit secondary antibody (universal polymer anti-mouse and rabbit Histofine® 1:400, Vector Laboratories, Burlingame, CA, USA) and streptavidin peroxidase followed by the peroxidase substrate diaminobenzidine tetrahydrochloride according to the manufacturer’s instructions. Negative control slides were incubated with normal rabbit serum and the secondary antibody alone. For dual-fluorescence immunostaining, the cross-sections were preincubated with 2 % casein in PBS followed by anti-GFP antibody (1:50, ab6556, Abcam Inc., Cambrigde, MA, USA) and anti-von Willebrand factor (1:50, ab6994, Abcam Inc., Cambrigde, MA, USA) overnight at 4 °C. The sections were subsequently incubated for 90 min with the secondary antibody anti-rabbit Alexa fluor® 488 (1:200, Life Technologies), anti-mouse Alexa fluor® 555 (1:200, Life Technologies) and 4’,6-diamidino-2-phenylindole (DAPI) (D3571, 1:100, Invitrogen, Life Technologies) to identify cell nuclei. The number of rASC-GFP^+^/vWF^+^ in which green fluorescence for GFP colocalized with red fluorescence for vWF and blue fluorescence for DAPI were counted from eight randomly selected microscopic fields for each tissue section under a 200× objective. The total tissue sections from six animals in each group were used to measure the final cell number.

### Measurement of angiogenesis, proliferation and apoptosis

Sections that contained mature ovarian follicles were immunostained to measure angiogenesis using tissue VEGF expression (SANT-SC-152, 1:100, Santa Cruz Biotechnology), proliferation using Ki67 expression (Anti-human monoclonal antibody mib-1 m7240, 1:100, Dako), and apoptosis using cleaved-caspase-3 expression (SANT-SC-1226, 1:100, Santa Cruz Biotechnology, Inc. Santa Cruz, CA, EUA) and the terminal deoxynucleotidyl transferase (TdT)–mediated dUTP nick-end labeling (TUNEL) assay. The ovarian sections were prepared as previously described and counterstained with hematoxylin. The tissue slices for the TUNEL assay were stained using a commercially available kit (In Situ Cell Death Detection Kit, Fluorescein, Roche, Berlin, Germany, 11684795910) following the manufacturer’s instructions. The primary antibody was omitted for the negative controls in each different immunohistochemistry staining.

Images of the sections were obtained using an image acquisition software system (Leica DM2500), and measurements were made using Leica QWin V3 software. A positive cell staining assessment was performed in eight different fields per animal at 200× magnification and the results are expressed as a percentage of the positive area (arbitrary unity/mm^2^). A red-brown coloring of the cytoplasm/nucleus of the granulosa cells was specified as positive staining (otherwise as negative staining). Two independent investigators blinded to the experimental treatments performed all measurements.

### Quantitative PCR

Total RNA was extracted from topic or grafted ovaries using the QIAzol Lysis Reagent and the RNeasy Micro Kit (Qiagen, Hilden, Germany) according to the manufacturer's instructions. The solution was treated with the RNase-Free DNase Set (Qiagen, Hilden, Germany). The total RNA obtained from each sample was quantified spectrophotometrically (ND100 NanoDrop®-Thermo Fisher Scientific Inc. Co.), and the RNA integrity was assessed by electrophoresis on a 1 % agarose gel.

Total RNA (1 μg) purified from each sample was transcribed into cDNA via reverse transcription using the RT^2^ First Strand Kit (Qiagen, Hilden, Germany) according to the manufacturer’s instructions. The synthesized cDNA underwent reaction in qPCR in PCR array plates (RT^2^ Profiler PCR Arrays, cat. PARN-404Z, QIAGEN-SABiosciences Corporation, USA) and the 7500 Real-Time PCR System (Applied Biosystems, CA, USA) were used. VEGFA (Catalog# PPR06748C; NM031836), Tgfβ (Catalog# PPR06430B; NM021578), Bcl2 (Catalog# PPR06577B; NM016993), and Egf (Catalog# PPR43509B; NM012842) were analyzed. The qPCR results were calculated using the ΔΔCT method and specific SABiosciences software. The gene expression results are provided as fold changes, relative to the reference group (Topic + vehicle). The software assign for this group (Topic + vehicle) a value of 1, due to ΔΔCT relative expression analyses method [46]. So, none value of fold expression lower than 1 might be considered significant. To enhance the biological significance of the gene expression, we stablished a cut off of values >3 and <3. Genes with fold regulation >3 were considered upregulated and those with values <3 were considered downregulated. The values were obtained for statistical analyzes, but relative expression reflects the number of times that a specific gene was expressed comparing to a reference sample or group and a significant value was considered to be a three-fold change in relative to Topic + vehicle group. All gene expression levels were normalized using the average of the housekeeping genes (Actb, B2m, Hprt1, Ldha, and Rplp1) following the software manufacturer’s instructions. Data were analyzed using Web Basis Data Analysis at http://www.sabiosciences.com/pcr/arrayanalysis.php.

### Statistical analysis

According to the Shapiro-Wilk normality test, unpaired *t*-test (for normal distribution) or Mann–Whitney tests (for non-normal distribution) were utilized to compare treatment groups (Topic + vehicle vs. Topic + rASC-GFP^+^ and Graft + vehicle vs. Graft + rASC-GFP^+^). Topic groups were compared with each other and the same was done for transplanted groups. The results were expressed as the mean ± standard deviation of the mean (unpaired *t*-test) or median (Mann-Whitney tests). All statistical analyses were performed using Graphpad Prism 5.0 (Graphpad Software Inc, CA, USA). *P* values lower than 0.05 were considered significant.

## Results

### rASC therapy does not interfere with ovarian morphology, follicular pool or tissue viability

The non-absorbable stitch helped to identify the ovarian grafts during euthanasia, and 100 % of the grafts were recovered. The grafts were enwrapped in adipose tissue, and some grafts exhibited a surrounding neovascularization network, which was visible to the naked eye.

**Fig. 2:**
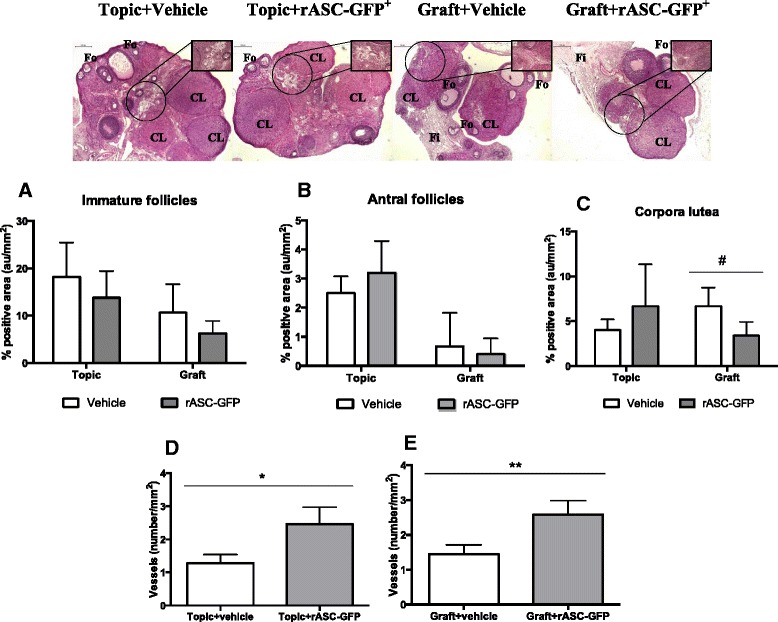
Representative photomicrographs of topic or freshly grafted ovarian tissue submitted to treatment with rASC-GFP^+^ or vehicle. HE Scale bar 500 μm. Zoom indicates area where blood vessels were counted (Scale bar 50 μm). Fo = Follicles. CL = Corpora Lutea. Fi = Fibrosis. Bottom figures depict ovarian follicle density (mean) of immature follicles (**a**), antral follicles (**b**) and corpora lutea (**c**) (Data shown in mean ± SD in **a** and **c** and median-interquartile range in **b**. #*p* = 0.0411. Unpaired *t* test) and blood vessel count (number/area) in Topic (2D) and Graft ovaries (2E) (*p = 0.0106. **p = 0.0019. Unpaired *t* test)

Morphologically, there were no difference in ovaries with no treatment or treated with vehicle (extra group). Ovarian follicles in both groups of topic ovaries at several maturation stages (primordial, primary, preantral and antral) were observed, and countless corpora lutea, most whole but some degenerated, were also observed. There was no difference with respect to treatment (rASC-GFP^+^ or vehicle) (Fig. 2).

A smaller concentration of ovarian follicles, corpora lutea, interstitial cells, and ovarian stroma was observed in both groups of transplanted ovaries in addition to the surrounding fibrosis and inflammatory infiltrate. However, there were ovarian follicles at several maturation stages and the whole corpora lutea. Overall, the rASC-GFP^+^ did not interfere with the ovarian morphology in either group (Fig. 2). There were no significant differences in the number of immature (topic *p* = 0.3168; graft *p* = 0.2386) and antral follicles (topic *p* = 0.1823; graft *p* = 0.6652) with rASC-GFP^+^ however a reduction in corpora lutea in the grafted group was observed (topic *p* = 0.2498; graft *p* = 0.0411) (Table 1; Fig. 2a–c).

**Table 1:** Ovarian follicles density according to treatment with adipose tissue-derived stem cells from transgenic rats for green fluorescent protein (rASC-GFP^+^), in topic and autologous grafted ovary

	Topic		Graft	
	Vehicle	rASC-GFP	Vehicle	rASC-GFP
Immature^a^	18.2 ± 7.2	13.8 ± 5.6	10.6 ± 6	6.2 ± 2.6
Antral^b^	2.5(2.0–3.0)	3.0(2.8–3.5)	0.0(0.0–1.5)	0.0(0.0–1.0)
Corpora lutea^a^	4.0 ± 1.2	6.7 ± 4.7	6.7 ± 2.1	3.4 ± 1.5*

Topic vehicle vs. Topic rASC-GFP^+^; Graft Vehicle vs. Graft rASC-GFP^+^. *p = 0.0411 vs. Vehicle. Data were shown in mean ± SD for Student *t*-test (^a^) or median-interquartile range for Mann-Whitney sum test (^b^), in according to the Shapiro-Wilk normality test

### rASC therapy induces an earlier resumption of the estrous phase in the transplanted group

All animals resumed cycling after transplantation, and the transplanted animals treated with rASC-GFP^+^ exhibited an earlier resumption of the estrous phase compared with the respective controls (Topic + vehicle: 7.1 ± 2.1 days vs. Topic + rASC-GFP^+^: 6 ± 1 days, *p* = 0.2615; Graft + vehicle: 11.6 ± 1.5 days vs. Graft + rASC-GFP^+^: 9.2 ± 1.9 days, *p* = 0.0475). The rats in the topic groups exhibited regular estrous cycles of four to five days, but the rats in the grafted groups exhibited significantly decreased numbers of estrous cycles with increased durations of the diestrus phase.

### rASC therapy does not interfere with estradiol levels both in topic and grafted ovaries

Serum estradiol levels 30 days after treatment were similar both in topic (Topic + vehicle: 6.43 ± 3.5 pg/ml vs. Topic + rASC-GFP^+^: 8.1 ± 3.4 pg/ml) and grafted (Graft + vehicle: 6.55 ± 2.27 pg/ml vs. Graft + rASC-GFP^+^: 8.2 ± 5.05 pg/ml) groups (*p* > 0.05).

### rASC therapy promotes an increase in VEGF gene expression and vessels in topic and grafted ovaries

**Fig. 3:**
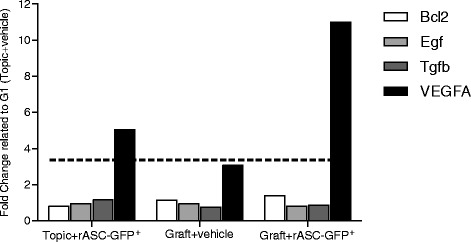
Gene expression of topic or grafted ovarian tissue submitted to treatment with rASC-GFP^+^ or vehicle, evaluated using qPCR. Each bar represents the value of fold change compared with the control group (Topic + vehicle). A significant value was considered over a three-fold change (dashed line). Data analyzed using Web Basis Data Analysis at http://www.sabiosciences.com/pcr/arrayanalysis.php

**Fig. 4:**
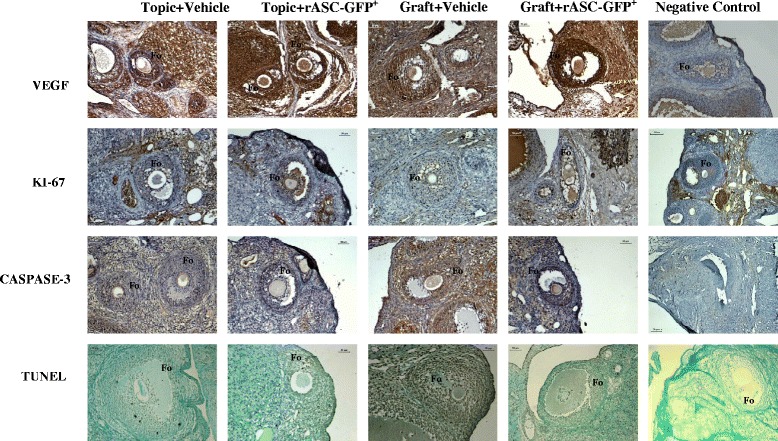
Photomicrographs of immunohistochemistry analysis for VEGF, Ki-67, Caspase-3 and TUNEL in ovarian follicles according to treatment with rat adipose tissue derived stem cells transgenic for green fluorescent protein (rASC-GFP^+^) or vehicle, in topic or freshly grafted ovary. A red-brown coloring of the cytoplasm/nucleus of the cells was specified as positive staining. For the negative controls, the primary antibody was omitted in each different immunohistochemistry staining. Treatment with rASC-GFP^+^ enhanced VEGF expression in topic ovarian tissue. Scale bar 50 μm. Fo = Follicles. VEGF = Vascular endothelial grown factor. TUNEL = terminal deoxynucleotidyl transferase (TdT)–mediated dUTP nick-end

The rASC-GFP^+^ injection significantly increased VEGF-A gene expression in both injected groups (Topic + rASC-GFP^+^ and Graft + rASC-GFP^+^ were 5- and 11-fold higher, compared with Topic + vehicle) (Fig. 3) and these groups had a greater number of blood vessels compared with their respective controls (Topic + vehicle and Graft + vehicle) (Fig. 2d–e). However VEGF labeling was increased only in topic ovaries (Topic + rASC-GFP^+^) in the immunohistochemistry assay (Table 2, Fig. 4).

**Table 2:** Immunohistochemistry analysis in ovarian follicles for angiogenesis (VEGF), cellular proliferation (Ki-67) and apoptosis (cleaved-caspase-3 and TUNEL), according to treatment with Adipose Tissue-Derived Stem Cells from transgenic rats for Green Fluorescent Protein (rASC-GFP), in topic and autologous grafted ovary

	Topic		Graft	
	Vehicle	rASC-GFP	Vehicle	rASC-GFP
VEGF	13.58 ± 4.4	27.13 ± 11.1*	25.46 ± 9.4	41.9 ± 11.7
Ki67	2.46 ± 0.55	2.12 ± 0.7	2.44 ± 1.5	1.72 ± 0.38
Caspase-3	4.23 ± 2.5	6.39 ± 2.9	6.97 ± 5.6	6.18 ± 3.44
TUNEL	0.03 ± 0.03	0.04 ± 0.08	0.13 ± 0.12	0.04 ± 0.05

Data are shown as percentage of positively stained follicle cells per area (arbitrary unity/mm^2^). *VEGF* Vascular Endothelial Grown Factor, *TUNEL* Terminal deoxynucleotidyl transferase (TdT)-mediated dUTP nick-end

Data were shown in mean ± SD; Student *t*-test; *p = 0.0352 vs. Vehicle

On the other hand, the rASC-GFP^+^ injection didn’t increase the expression of genes involved in cellular proliferation (EGF and TGFβ1) and apoptosis (Bcl2) (Fig. 3). In agreement with these findings, apoptosis (TUNEL and caspase-3) and proliferation (ki67) rates in the immunohistochemistry assay were also similar between the groups (*p* > 0.05) (Table 2, Fig. 4).

**Fig. 5:**
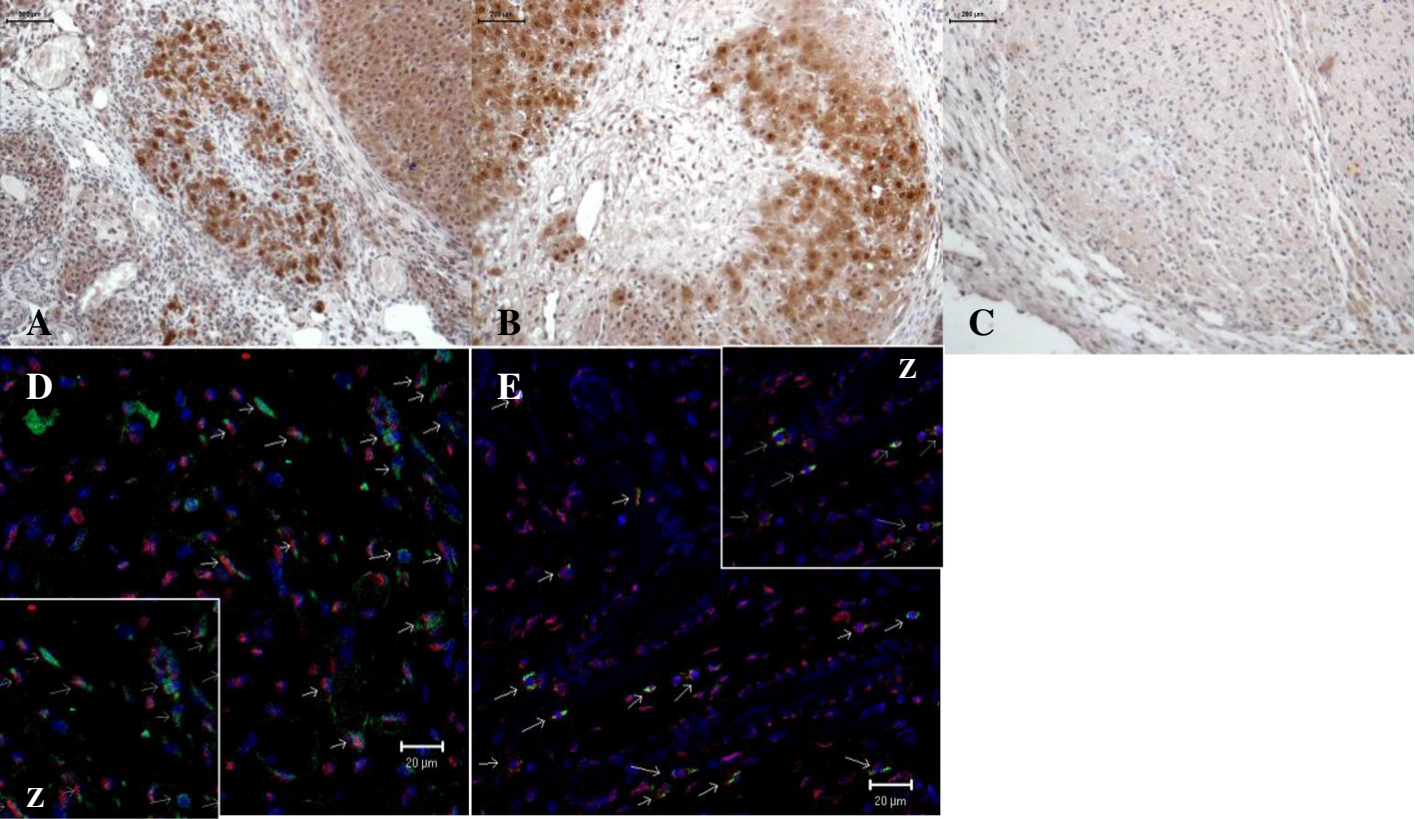
Photomicrography of rASC-GFP^+^ identification in topic (**a**) and grafted (**b**) ovaries marked in dark brown. In (**c**) negative control. Note that the cellular morphology was similar in both groups. Scale bar 200 μm. Immunofluorescence of rat ovarian sections with double labeling for GFP^+^ and von Willebrand Factor (vWF) in topic (**d**) and freshly grafted (**e**) ovaries. The arrows indicate rASCs that co-express GFP (*Green*) and VWF (*red*). Cell nuclei are shown in blue (DAPI). Scale bar 20 μm. *z* = zoom 2×

rASC-GFP^+^, identified by immunohistochemistry, were observed only in the ovarian stroma, and a similar number of cells in both injected groups were observed (Topic + rASC-GFP^+^: 176.5 ± 85.4 vs. Graft + rASC-GFP^+^: 147 ± 65.8, *p* = 0.6041) (Fig. 5a–b). Interestingly, double immunostaining for GFP and vWF demonstrated a colocalization of the two markers in some clusters of ASCs in Topic + rASC-GFP^+^and Graft + rASC-GFP^+^. vWF is a marker of the endothelial phenotype, and these data suggest that a fraction of the rASCs could behave as endothelial cells (Fig. 5d–e).

## Discussion

Our results demonstrated that cell therapy with rASC in fresh ovarian grafts was safe and effective as evidenced by the morphological and immunohistochemical parameters. The results also indicated an early resumption of estrous cycle.

We chose in situ injection of rASC-GFP^+^ because previous studies have shown a massive cell loss following intravenous injections [37]. No deformity, tumor formation, or atrophy was observed in the rASC-GFP^+^ rats, indicating that the injection had no side effects. The number of cells used in the literature has widely varied [36–38]. One group with normal (topic) ovaries and another group with grafted ovaries were used in the current study because all previous studies in rats used chemotherapy-induced ovarian damage. We used a concentration of 5 × 10^4^ cells/ovary (1 × 10^5^ cells/animal), which was the lowest concentration used in previous studies [36].

rASC-GFP^+^ were only found in the ovarian stroma, not in follicular cells, which was described by other investigators in experimental models of chemotherapy-induced ovarian lesion [35, 36]. However, the reason for this limited localization is unknown. A higher vascularization of the stroma may, in part, explain this finding. The morphological results of the rASC-GFP^+^ group did not exhibit atypical or irregular cells. This finding suggests that this therapy is safe. Cell injections did not produce changes in ovarian morphology at either site. Group differences, such as a smaller number of ovarian follicles and the presence of fibrosis, were observed, but adaptive mechanisms inherent in the transplantation process [17, 18, 43, 44] were likely the reason, not effects from the treatment (i.e., the injection of rASC-GFP^+^). Therefore, the treatment can be considered to be effective and stable in fresh ovarian tissue, which differs from what has been previously observed in the heart, in which very few cells remained at the injection site [28, 29, 34]. In this study, rASC-GFP^+^ settle in the ovarian tissue after direct injection (Topic + rASC-GFP^+^and Graft + rASC-GFP^+^). The groups exhibited similar morphology, and the evidence indicates that rASC-GFP^+^ remain active in the ovarian stroma (both topic and grafted) to promote the expression of pro-angiogenic factors, such as VEGF and vWF.

The previously described finding does not strongly support the idea that engrafted cells differentiate into functional vascular cells (transdifferentiation) or stem cells that fuse with other local cells. The mechanisms involved in stem cell-based therapy are not well understood. Engrafted stem cells can produce encouraging paracrine signals for cell survival in the ischemic environment [27, 45], and the hypoxic microenvironment of the ovarian tissue may influence this type of action mechanism. Our finding demonstrated that this therapy was associated with an early functional resumption of the grafts, as demonstrated by vaginal smear cytology. Further studies are needed to better assess cells that express the endothelial phenotype because of the influence of a hypoxic environment. On euthanasia day (30 days after operative procedures), serum estradiol levels were similar in both groups (topic or grafted) despite the treatment with rASC-GFP. We didn’t colleted the blooda samples in the first days after operative procedures. It may be a limitation to interpret these data. As in animals treated with RASC-GFP^+^ exhibited early functional return of the estrous cycle, estradiol serial measurements could determine the endocrine profile of this graft.

Human ASCs secrete a variety of growth factors [34]; however, we focused on VEGF, which is known to affect follicular growth in ovaries and is stimulated under conditions of hypoxia [34], similar to the conditions present following transplantation. In our study, VEGF-A gene expression was more pronounced in both groups treated with rASC-GFP^+^, which may have led to an increase in the number of blood vessels compared with the controls. However, VEGF tissue expression was enhanced only in the topic ovary treated with rASC-GFP^+^, a finding that might be related to the late evaluated period after the transplant (30 days).

Another interesting finding was the apparent equilibrium between proliferation and apoptosis in the tissue. There was no increase in apoptosis- or proliferation-related gene expression (Bcl2, Tgfb, and EGF), which was also confirmed by immunohistochemistry. rASC-GFP^+^, most likely through the release of cytokines, prevented apoptosis (according to cleaved-caspase-3 and TUNEL) in both groups; however, Bcl2 expression did not change. These data support the previous evidence indicating that rASC-GFP^+^ show beneficial effects, such as the inhibition of apoptosis [47], inflammation [48], and other immune responses [49] and the ability to produce angiogenic factors under hypoxia [50, 51].

In our study, the density of primordial and primary follicles was significantly higher in the non-grafted compared with grafted samples. These results clearly demonstrated that ovarian tissue transplantation led to follicle loss in agreement with the literature reports [17, 18, 43, 44].

In addition, rASC-GFP treatment reduced the number of corpora lutea in grafted ovaries; however, this finding may not be a result of rASC-GFP^+^ injection. Corpora lutea are not a useful indicator of ovarian activity because they can persist for up to 14 days. Thus three or more generations of corpora lutea could exists from the preceding ovulatory cycles [52].

## Conclusion

The present study suggested that: 1) rASC-GFP^+^ therapy does not interfere with ovarian morphology, follicular pool or tissue viability; 2) rASC-GFP^+^ remain viable and in similar amounts in both topic and grafted ovaries and have an endothelial-like phenotype (as indicated by double immunostaining for GFP and vWF); 3) rASC-GFP^+^ therapy promotes an increase in VEGF-A gene expression and the number of blood vessels in ovarian tissue; and 4) rASC-GFP^+^ therapy induced earlier functional resumption in grafted ovaries, as indicated by estrous vaginal smears. This pilot study may be useful in the future for new researches on frozen-thawed ovarian tissue.

## Additional file

Additional file 1:
**ASC isolation and ex vivo expansion.** (DOC 23 kb)

## Abbreviations

ASCs: Adipose tissue-derived stem cells
VEGF: Vascular Endothelial Growth Factor
qPCR: Quantitative gene expression
vWF: Von Willebrand Factor
DMEM: Dulbecco’s modified Eagle’s medium
DMSO: Dimethyl sufoxide
FBS: Fetal Bovine Serum
GFP: Green fluorescente protein
HE: hematoxylin and eosin
PBS: Phosphate-buffered saline
PO: Postoperative
TUNEL: Deoxynucleotidyl transferase (TdT)–mediated dUTP nickend labeling
