# Supplementary material for: Does adipose tissue-derived stem cell therapy improve graft quality in freshly grafted ovaries?
Source: Reprod Biol Endocrinol. 2015 Sep 23;13:108. doi: 10.1186/s12958-015-0104-2 (PMC4580300; doi:10.1186/s12958-015-0104-2)
Supplement: Additional file 1: — ASC isolation and ex vivo expansion. (DOC 23 kb) [file 12958_2015_104_MOESM1_ESM.doc]

**ASC isolation and *ex vivo* expansion**

The harvested tissue was dissociated by digestion with 0.075% type IA collagenase (Sigma-Aldrich, Inc.) for 45 minutes. Enzyme activity was stopped and the cell suspension was centrifuged at 300g for 15minutes. Pelleted cells were recovered and plated onto 10-cm culture plates (NUNC, Rochester, NY). At 24-hour intervals, cultures were washed with PBS to remove contaminating erythrocytes and other unattached cells, and then reefed with fresh medium. The plating and expansion medium consisted of low glucose Dulbecco’s modified Eagle’s medium (DMEM) supplemented with 10% Fetal Bovine Serum (FBS) and penicillin/streptomycin antibiotics (Invitrogen Corporation, Carlsbad, CA).

Cells were maintained at 37ºC with 5% CO_2_ in tissue culture dishes and fed twice a week until they reached 80% of confluence - usually within 5 to 7 days after the initial plating. Once 80% confluence was reached (passage 0), adherent cells were detached with 0.25% trypsin-EDTA (Vitrocel Embriolife, Campinas, SP, Brazil) and were either replated at 1x10^4^cells/cm^2^ or used for experiments. Cultures were passaged every 3 to 5 days and used for experimental procedures until passage 3.
